# Moral Psychopharmacology Needs Moral Inquiry: The Case of Psychedelics

**DOI:** 10.3389/fpsyt.2021.680064

**Published:** 2021-08-02

**Authors:** Nicolas Langlitz, Erika Dyck, Milan Scheidegger, Dimitris Repantis

**Affiliations:** ^1^Department of Anthropology, The New School for Social Research, New York, NY, United States; ^2^Department of History, University of Saskatchewan, Saskatoon, SK, Canada; ^3^Department of Psychiatry, Psychotherapy and Psychosomatics, University Hospital of Psychiatry Zurich, Zurich, Switzerland; ^4^Charité – Universitätsmedizin Berlin, Department of Psychiatry and Psychotherapy, Berlin, Germany

**Keywords:** psychedelics, hallucinogens, morality and values, extrapharmacological variables, anthropology, ethnography, history, psychotherapy

## Abstract

The revival of psychedelic research coincided and more recently conjoined with psychopharmacological research on how drugs affect moral judgments and behaviors. This article makes the case for a moral psychopharmacology of psychedelics that examines whether psychedelics serve as non-specific amplifiers that enable subjects to (re-)connect with their values, or whether they promote specific moral-political orientations such as liberal and anti-authoritarian views, as recent psychopharmacological studies suggest. This question gains urgency from the fact that the return of psychedelics from counterculture and underground laboratories to mainstream science and society has been accompanied by a diversification of their users and uses. We propose bringing the pharmacological and neuroscientific literature into a conversation with historical and anthropological scholarship documenting the full spectrum of moral and political views associated with the uses of psychedelics. This paper sheds new light on the cultural plasticity of drug action and has implications for the design of psychedelic pharmacopsychotherapies. It also raises the question of whether other classes of psychoactive drugs have an equally rich moral and political life.

## Introduction: A New Field Of Research

Since the 2000s, neuropsychopharmacologists have grown interested in the neurochemical foundations of moral behaviors. Experimental paradigms from social psychology and behavioral economics have been used to study the effects of drugs, for example how antidepressants such as citalopram enhanced harm aversion and affected people's inclination to retaliate against unfairness (1), how oxytocin affected one's readiness to trust others (2) and motivated both in-group favoritism and out-group derogation (3), how regular users of stimulants showed less prosocial behavior (4), or how MDMA and LSD increased emotional empathy and altruism (5). Analogous to moral psychology, a field that emerged at about the same time, this rapidly growing body of research gave birth to a new subfield that could aptly be called “moral psychopharmacology.”

## The Context Dependence of Psychedelics Varies Their Moral Effects

In the case of psychedelics, research on their moral effects is of particular importance. Politically, psychedelic drug use is likely to increase and take place in a variety of non-medical contexts as a number of jurisdictions introduce decriminalizing legislation concerning psychedelics. In May 2021, for example, Psilonautica and Drug Science, two UK-based organizations, revealed findings from a study showing that a majority of the British public support the medical use of psychedelics for trauma-based injuries and end-of-life anxiety. American cities like Denver, Oakland and Santa Cruz went further and decriminalized psilocybin, and in 2020 the state of Oregon decriminalized psilocybin and approved it for medical use. The legal prohibition on psychedelics is beginning to change, but its regulatory future remains context specific. Scientifically, the moral psychopharmacology of psychedelics is especially interesting because their effects have long been shown to depend crucially on extra-pharmacological factors. Since the 2010s, what is colloquially called “set and setting,” the subject's mindset and their social and physical environment, has attracted renewed interest in the revival of psychedelic research. Carhart-Harris et al. (6) argued that the drugs' 5HT_2a_ receptor agonism renders the psychedelic experience exceptionally sensitive to context, both internal and external. While common psychophysiological effects on serotonin-mediated neurobehavioral circuits may shape some of the cross-cultural similarities in patterns of psychedelic use and experiences, their cultural desirability is mostly shaped by political factors (7). What distinguishes research in this vein from research on the placebo effect is that, in the case of psychedelics, the effects of extra-pharmacological factors appear to be pharmacologically mediated and amplified. It is part of psychedelic drug action to blur the line between the pharmacological and the nonpharmacological, between drug action and social context.

This context-dependence is also of clinical importance as psychedelic-assisted therapy of depression and other psychiatric conditions combines pharmacotherapy with psychotherapy, actively mobilizing extra-pharmacological factors to improve treatment outcomes (even though ongoing randomized controlled trials do not explicitly focus on the psychotherapeutic contribution). The subjective experience shaped by set and setting might not just be epiphenomenal but causally related to the drugs' therapeutic effects (8). Although interactions with other people crucially affect how patients experience the effects of psychedelics, in medical research, it is often a mere afterthought that drug treatments of psychiatric patients have significant implications for their social relationships (9). Outside of a clinical setting, these contextual features will be even more difficult to assess, predict, or control.

This relationship to cultural context opens up a dimension of moral psychopharmacology directly relevant to clinical work, which cannot be guided only by considerations of the normal and the pathological that usually inform medical interventions. Thereby, the question of how drugs affect human relationships and what makes a *good* relationship comes into the purview of psychiatric drug treatment. In the clinical context, moral psychopharmacology moves from the limited ecological validity of behavioral economic games and moral dilemma experiments to issues that are highly relevant to the moral fabric of everyday life (even if the intricacies of romantic relationships or family life have not figured prominently in moral philosophy). In this respect, the moral psychopharmacology of *all* drugs that affect social behavior appears significant. Notably, recent studies highlight how psychedelics and empathogens modulate conscious decision-making and behavioral orientations in various psycho-socio-environmental domains (5, 10, 11). The pronounced context-dependence of psychedelics and their application in the context of different psychotherapeutic approaches or non-clinical uses gives particular urgency to the question of how they affect people's moral judgment and behavior. After all, the moral effects might not be one-dimensional, simply increasing or decreasing regard for others as concerns about moral “enhancement” and “degradation” imply [(12, 13), p. 233–35, 261–63]. Instead, the moral effects of psychedelics appear to differ depending on the context of their use and the mind-set of the user.

The successful mainstreaming of psychedelics through medical research has introduced these substances to populations beyond the so-called psychedelic research community. The growing diversity of users and uses broadens the range of extra-pharmacological factors that shape the drugs' moral effects. Historical and ethnographic research can complement contemporary neuropsychopharmacology studies with studies of cultural contexts that examine other sets and settings in relation to drug uses and effects. While members of the American counterculture used LSD, mescaline, and psilocybin as psychopharmacological tools to liberate individuals from the ills of their society, Huichol youth ingested peyote buttons to become full members of their own society (14, 15), and Native American Church worshippers consumed peyote to foster indigenous resistance to North American colonialism (16, 17). In the 1960s, psychedelics were taken to experience a mystical union that users claimed fostered a sense of universal love, but anthropologists have also described Amazonian societies that used them in rituals to prepare for violent intergroup conflict (18–20). At present, psychedelic therapists and progressive intellectuals suggest that psychedelic experiences could help to work through the cultural trauma of racist discrimination (21, 22), and one study found that intense psychedelic experiences predicted liberal political views, openness, and nature relatedness, while negatively predicting authoritarian political views [(23); but see (24)]. At the same time, a budding traditionalist scene finds inspiration in the right-wing writer and psychonaut Ernst Jünger and, according to media reports, psychedelics might also play a role in the political radicalization of far-right groups in the United States (25–27). A moral psychopharmacology of psychedelics needs to explain how pharmacological and extrapharmacological factors interact to produce such seemingly contradictory varieties of psychedelic experience.

## Taking Psychedelic Research Beyond the Two-Culture Divide

A well-established but potentially misleading account of the moral and political versatility of psychedelics draws from an older naturalist ontology that suggests that there is one nature and many cultures that interpret it differently. It has inspired a neat division of labor between the disciplines: neuropsychopharmacologists study the effects of drugs on the brain, cultural anthropologists and historians study how different groups of humans make sense of them at different points in time. In recent decades, however, both the ontological and the epistemological divide between the two cultures of the natural sciences on the one hand and the humanities and interpretive social sciences on the other hand have been called into question from very different angles. Culture is no longer the exclusive domain of the humanities and human sciences but has become an object of research in cultural neuroscience and cultural primatology (28, 29). The same is true for related ontological categories such as the mind, which used to organize the disciplinary divide between *Geistes-* and *Naturwissenschaften* in the German-speaking world but is now the subject matter of evolutionary and brain research. This new naturalism has inspired research programs in the social sciences that have sought to break down the barrier between the natural and the human by presenting the scientific objects of the natural sciences as constructed by humans (30–32). While these challenges to the dichotomies of nature/culture, nature/human, or mind/matter might not be equally compelling across all areas of study, they are germane to a class of psychoactive drugs whose effects depend on their users' personalities, expectations, and cultural beliefs as well as the social setting in which they are ingested. Studying the moral effects of psychedelics raises epistemological challenges that extend across the divide between the natural sciences and the humanities (33).

More recently, extra-pharmacological factors have attracted attention in psychedelic research, which again stimulates interdisciplinary debates and transdisciplinary collaborations (34, 35). The experiential diversity of psychedelic states calls for novel approaches combining phenomenological methods and empirical research to better understand the structure and dynamics of altered states of consciousness that go beyond their conceptualization as “brain states”. Social anthropologist David Dupuis, clinical psychologist Rosalind Watts, and neuroscientist Christopher Timmermann propose moving beyond research that correlates first-person reports and third-person measurements of brain activity and attend to the second-person dimension of what they call “psychedelic apprenticeship” (36). Their focus on the social mediation of psychedelic-induced experiences can account for why psychedelics have turned out to be a “double-edged sword” that can either benefit or harm their users—or even do both at the same time. Psychedelics have been shown to increase suggestibility while concealing this influence of others by endowing visions with a noetic quality, a deep sense of having obtained unmediated knowledge that requires no external validation or evidence (37–39). This more variable set of outcomes opens up the possibility of harnessing psychedelic apprenticeships to different therapeutic goals and ethical projects. Subjects undergoing drug-induced mystical experiences might even be steered toward religious conversion and New Age spiritualities—a prospect that has raised concern among psychedelic psychotherapists who seek to secularize the field and worry about the abuse of power on the part of guides and therapists (40, 41).

The context sensitivity of psychedelics entails that randomized placebo-controlled trials are insufficient for appreciating the full range of their possible effects, nor do they capture the diverse ways in which consumers may encounter psychedelics in medical and non-medical settings alike. If the pharmacological activity of a drug changes the relationship between a living thing and its environment in ways that depend on the living thing's mindset and the particular quality of the environment, then the environment would be inscribed in the observed pharmacological effect—even if the placebo effects were subtracted from the effects of the verum. In the 1950s, anthropologist Anthony Wallace had already noted that Euro-American subjects who had received mescaline in the laboratory reported very different psychotropic effects than Native Americans who had eaten peyote buttons in religious ceremonies. At a time when placebo-controlled trials only just emerged as the gold standard of pharmacological research, Wallace proposed to complement them by culture-controlled trials that tested the same drug at the same dosage under different social and cultural circumstances [(13), p. 116–26, 185–92; (42)]. Reanimating this largely forgotten approach today would provide moral psychopharmacology with an opportunity to import experimental paradigms from social, moral, and political psychology.

## “Upstream” Design of New Uses of Psychedelics

The ethical plasticity of psychedelic-induced experiences poses a formidable bioethical problem. Usually, bioethics offers a moral evaluation of the social consequences of new biotechnologies or pharmaceuticals. For example, debates over antidepressants or so-called cognitive enhancers assumed that the drugs have certain effects established by clinical observation and psychopharmacological experiment, while bioethicists evaluated the social consequences of these effects downstream from pharmacology and proposed how to regulate the drugs' applications. A growing body of bioethical literature on psychedelics evaluates the use of psychedelics to neurochemically enhance the sense of meaning in psychedelic-assisted psychotherapy and their use to develop virtues, suppress vices, and enhance moral behavior (43, 44). What distinguishes the notion of psychedelic moral enhancement, as advocated by some authors, from traditional notions is that it recognizes that amplifying particular aspects of prosociality produces trade-offs and adverse effects. For example, MDMA boosts prosocial behavior toward members of one's in-group but not toward the outgroup, which can result in a redistribution of limited resources (45). However, if psychedelics reduced their users' sense of self-importance, they might free up resources in favor of both neighbors and strangers, increasing other-regard solely at a cost to egocentric motivations (46, 47).

This model of bioethics presupposes conceptions of what counts as moral and measures technologies against them. It is based on a separation of technology and morality, means and ends. However, psychedelics present a case where the means transform the ends. Although a recent trial by Carhart-Harris et al. comparing psilocybin and escitalopram in long-standing, moderate-to-severe major depressive disorder did not show significant difference in antidepressant effects, it produced secondary effects on qualitative dimensions of psychosocial functioning that transformed patients' attitudes toward themselves, others, and the world (48). Notably, patients from other trials undergoing conventional treatment for depression reported that medications and short-term talk therapies tended to reinforce their sense of disconnection and avoidance, whereas psychedelic treatment encouraged connection and acceptance or a sense of “reconnecting” with past values that had faded over the years (49–51). Of course, these experiential reports leave open whether people actually reconnect with their own values, what role their heightened suggestibility plays in this process, and whether the pharmacological modulation of affective relations to the world and to others privileges certain moral and maybe even political transformations over others. Do different psychedelic compounds produce different moral effects and ethical orientations? How do psychedelics compare with other classes of psychotropic drugs? Whatever the mechanism, variability, and scope of such pharmacological transvaluation of values, it led psychiatrist William Smith and medical ethicist Dominic Sisti to call for an enhanced consent process acknowledging that patients might experience significant shifts in their ethical outlook and worldview, which they cannot fully foresee from the perspective of their pre-therapeutic self (52). The challenge is to invent new research practices that observe and reflect on the combining of neurochemistry and morality in the laboratories and clinics where this amalgamation is happening.

Another challenge involves observing how psychedelics are used beyond the laboratory and the clinic, turning psychopharmacology into a field science by adopting practices from ethnopharmacology, ecology, and other areas of field biology. For instance, battery-powered EEG equipment already allows one to study in naturalistic settings how ayahuasca, an Amazonian jungle environment, guidance by experienced ayahuasceros, and other variables concur in their effect on brain waves, and placebo-controlled field experiments seek to tease apart the impact of pharmacological and extra-pharmacological factors in such an ayahuasca ceremony (53, 54). Other field sciences such as cultural primatology that use recent advances in statistics to explain how complex interactions between large numbers of ecological factors shape animal behavior might provide further methodological inspiration for how to study the interplay between pharmacological and extrapharmacological factors [e.g., (29, 55), p.180–90; (56)].

Finally, the challenge is to study historical uses of psychedelics as natural experiments that shed light on the context dependence of the psychedelic experience. From a regulatory perspective, it will still largely depend on randomized placebo-controlled trials, whether psychedelics will have a wide or a restricted role as prescription medicines. The goal is not to replace but to complement those trials, as a wide range of contextual variables are likely to determine the therapeutic benefits of psychedelics. An upstream approach to the morally transformative effects of psychedelics places psychopharmacologists, psychiatrists, social researchers, and humanities scholars side by side to study, assess, and redesign psychedelic experiences in their social, cultural, and historical contexts (57, 58). Ideally, laboratories and clinical trial sites would embed anthropological, sociological, and philosophical observers instead of keeping them cordoned off in their respective university departments. Ethnographic descriptions and comparisons of the uses of psychedelic drugs at different sites, including non-medical sites, could be fed back into the fashioning of new uses. First-hand familiarity with research and therapeutic practices as well as everyday personal contact enable members of different disciplines to inform each other's thinking and devise approaches that make good use of the context dependence of psychedelics in light of ongoing discussions of what constitutes a *good* use.

## Conclusion: Extending Research and Development into the Extra-Pharmacological Realm

If moral psychopharmacology took it upon itself to develop forms of psychedelic apprenticeship for the currently sprawling medical and non-medical applications of psychedelics, it would extend pharmaceutical research and development into the extra-pharmacological realm. Such a design process needs to be informed by best practices in clinical psychology and cognate fields, but, intellectually, it cannot hide behind professional prescriptions because what counts as good and bad is precisely what is at stake here. It is an open philosophical question that has to be answered in a recursive process of psychopharmacological experimentation, clinical and ethnographic observation, historical research, and ethical reflection. The evaluation of new uses of different drugs in the laboratory, the clinic, and in the wild should not be confined to the armchair, removed from these spaces and the experiences they engender. That is why research and development of psychedelics in context also requires research and development of knowledge cultures that bridge the gap between neuropsychopharmacology, social research, and the humanities. It is an empirical question whether such a blending of moral psychopharmacology with moral inquiry would remain confined to psychedelics because of their peculiar pharmacological properties, or whether it could become a model for working with other classes of psychotropic substances as well.

## Data Availability Statement

The raw data supporting the conclusions of this article will be made available by the authors, without undue reservation.

## Author Contributions

NL, DR, MS, and ED contributed to conception and design of the manuscript. NL wrote the first draft of the manuscript. DR, MS, and ED wrote sections of the manuscript. All authors contributed to manuscript revision, read, and approved the submitted version.

## Conflict of Interest

The authors declare that the research was conducted in the absence of any commercial or financial relationships that could be construed as a potential conflict of interest.

## Publisher's Note

All claims expressed in this article are solely those of the authors and do not necessarily represent those of their affiliated organizations, or those of the publisher, the editors and the reviewers. Any product that may be evaluated in this article, or claim that may be made by its manufacturer, is not guaranteed or endorsed by the publisher.
